# Leiomyoma mimicking an incarcerated inguinal hernia: A rare complication of laparoscopic hysterectomy

**DOI:** 10.4103/0972-9941.78351

**Published:** 2011

**Authors:** Carlos Apestegui, Saadallah Tamer, Olga Ciccarelli, Eliano Bonaccorsi-Riani, Etienne Marbaix, Jan Lerut

**Affiliations:** Department of Abdominal and Transplantation Surgery University Hospitals Saint-Luc, Université Catholique de Louvain - UCL, 1200 Brussels-Belgium; 1 Department of Abdominal and Transplantation Pathology, University Hospitals Saint-Luc, Université Catholique de Louvain - UCL, 1200 Brussels-Belgium

**Keywords:** Cellular spilling, inguinal hernia, laparoscopic surgery, leiomyoma

## Abstract

A 52-year-old, obese, female patient was referred for a right inguinal mass, which appeared seven months after a laparoscopic hysterectomy, which was performed because of myomatosis. Despite several examinations, including ultrasound, computed tomography (CT)-Scan, positron emission tomography (PET)-CT, and ultrasound-guided biopsy, the diagnosis remained unclear until surgical exploration, which disclosed a well-encapsulated solid tumour corresponding to a fibrotic leiomyoma. Spilling of leiomyoma cells is a rare and unusual complication of laparoscopic surgery. Tumour development in the inguinal canal after laparoscopic gynaecological surgery should be kept in mind in the differential diagnosis of inguinal hernia and other uncommon pathologies.

## INTRODUCTION

Minimally invasive procedures, especially myomectomy and hysterectomy, are increasingly performed in gynaecological surgery. New technical developments may bring about new complications in daily clinical practice. We report a case of implantation of a myoma in the inguinal canal following laparoscopic hysterectomy.

## CASE REPORT

A 52-year-old woman was referred to our department by her gynaecologist because of the development of a right inguinal tumour. Questioning and clinical examination revealed morbid obesity (BMI > 42), hypertension, tubal ligation, and resection of breast adenoma. In April 2009 a laparoscopic total hysterectomy was performed because of severe menorraghia caused by leiomyomatosis. An ultrasound examination showed that the uterus measured 99 × 60 mm; in addition, several subserosal and intramural leiomyomas were visualised.

Total radical laparoscopic hysterectomy was performed following the usual technique, using an open umbilical approach and insufflation of three liters of CO2. The morcellated uterus and both ovaries were removed transvaginally. Macroscopic and microscopic pathological examinations confirmed the presence of an intramural haemangioma and two submucosal myomas with diameters ranging from 1.5 to 5 cm.

**Figure 1:**
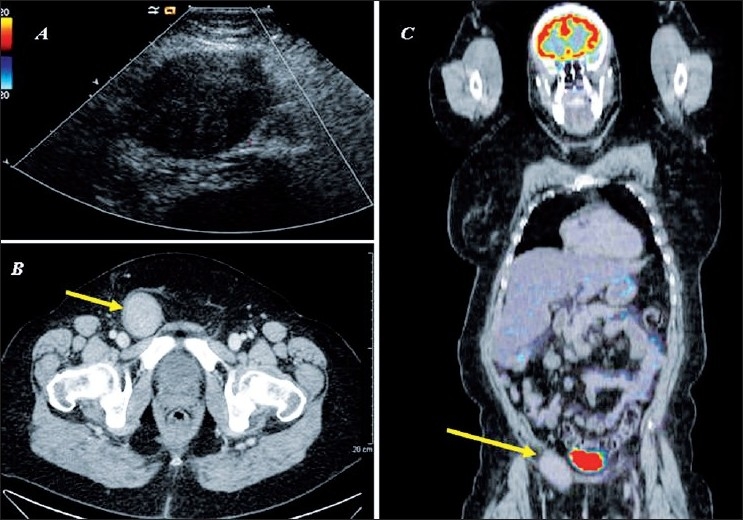
(A) Ultrasound showing a 6.3 × 4.2 cm hypoechogenic, nonvascularised inguinal tumour. (B) CT scan showing a 4 × 6 cm, hyperdense vascularised tumour. (C) PET-CT scan: there is no uptake of labeled glucose in the inguinal canal

Three-and-a-half-months later, she discovered a right inguinal mass. An ultrasound examination revealed a 6.3 × 4.2 cm hypoechoic, non-vascularised tumour, possibly corresponding to a hematoma [Figure 1A]. Three months later, an ultrasound examination showed the presence of a heterogeneous, vascularised, hypoechoic tumour, compatible with an inguinal lymph node or hernia. A contrast CT-scan carried out three weeks later revealed a 4 × 6 cm, hyperdense vascularised tumour suggesting a sarcomatous lesion [Figure 1B]. However, the PET-CT scan suggested a benign tumour, as there was absence of metabolic activity [Figure 1C]. Ultrasound-guided biopsy, performed six days later, was inconclusive due to insufficient tissue sampling.

Surgery was finally proposed in order to exclude an incarcerated inguinal hernia. In November 2009, seven months after the hysterectomy, and four months after the onset of symptoms, the patient underwent surgical exploration. After opening the fascia of the greater abdominal oblique muscle, a vascularised tumour, situated towards the middle of the round ligament, was identified. The tumour was removed and a Shouldice hernia repair was carried out, because of the presence of a small direct hernia. Her recovery was uneventful.

**Figure 2:**
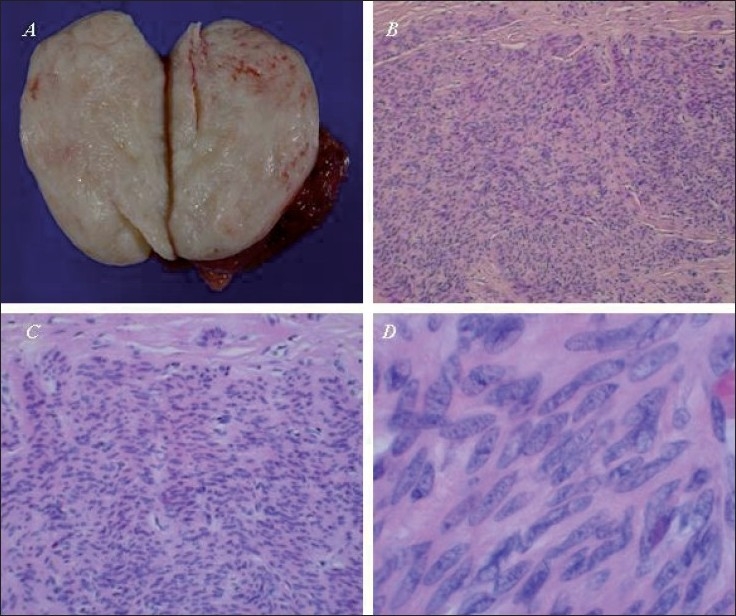
(A) Macroscopic aspect of the inguinal tumour specimen. (B/C/D): H and E staining (4×, 10×, 40×) confi rms the diagnosis of a fi brotic leiomyoma

Macroscopic examination of the specimen revealed a 6.5 × 4 × 4.5 cm, white-coloured, well-encapsulated, solid tumour. Microscopy revealed typical features of a fibrotic, benign leiomyoma. Immunohistochemistry confirmed the diagnosis, as the activities of muscular desmine and actin were elevated. [Figure 2A–D].

## DISCUSSION

Laparoscopic hysterectomy is a common indication of minimally invasive gynaecological surgery. Indications and complications of this procedure have been well-documented.[1] The five-year-recurrence rate of leiomyoma after laparoscopic resection is reported in up to 16.7% of the patients and the time lapse between surgery and recurrence ranges from four to ninety-five months.[2] Aberrant tumour implantation and dissemination of leiomyoma have been very rarely reported. Cellular spilling during laparoscopic surgery can be explained by different mechanisms such as direct cell implantation in trocar wounds, ‘unprotected’ tumour extraction, contamination of instruments and gaseous turbulence, embolising cells into different sites.[3] Aberrant implantation and subsequent leiomyoma growth in the abdominal wall and in the peritoneum have been reported.[4–5] We report, to the best of our knowledge, the first case of implantation of a leiomyoma in the inguinal canal. This condition can be easily confounded with an incarcerated inguinal hernia, especially in women. The hypothesis put forward is that the observed condition was favoured by spilling of cells due to the morcellation of the uterus (before transvaginal extraction was performed) in the presence of a pneumoperitoneum.

In conclusion, migration and implantation of tumour cells is a rare complication of laparoscopic surgery for uterine myoma. Depending on the mechanism of spreading, tumour implantation can occur at various locations. Laparoscopic surgeons and gynaecologists should be aware of this possibility during the follow-up of patients undergoing such a procedure. Aberrant tumour implantation should be taken into consideration in the differential diagnosis of any pathology of the inguinal canal.

## References

[1] Seinera P, Arisio R, Dedko A, Farina C, Crana F (1997). Laparoscopic myomectomy: Indications, surgical technique and complications. Hum Reprod.

[2] Doridot V, Dubuisson JB, Chapron C, Fauconnier A, Babaki-Fard K (2001). Recurrence of leiomyomata after laparoscopic myomectomy. J Am Gynecol Laparosc.

[3] Targarona E, Martinez J, Nadal A, Balagué C, Cardesa A, Pascual S (1998). Cancer dissemination during laparoscopic surgery. World J Surg.

[4] Wada-Hiraike O, Yamamoto N, Osuga Y, Yano T, Kozuma S, Taketani Y (2009). Aberrant implantation and growth of uterine leiomyoma in the abdominal wall after laparoscopically assisted myomectomy. Fertil Steril.

[5] Kumar S, Sharma JB, Verma D, Gupta P, Roy KK, Malhotra N (2008). Disseminated peritoneal leimyomatosis: An unusual complication of laparoscopic myomectomy. Arch Gynecol Obstet.

